# Serum contents of endocannabinoids are correlated with blood pressure in depressed women

**DOI:** 10.1186/1476-511X-11-32

**Published:** 2012-02-28

**Authors:** WS Vanessa Ho, Matthew N Hill, Gregory E Miller, Boris B Gorzalka, Cecilia J Hillard

**Affiliations:** 1 Department of Pharmacology & Toxicology, Medical College of Wisconsin, Milwaukee WI 53226, USA; 2 Department of Psychology, University of British Columbia, Vancouver V6T 1Z4, BC, Canada; 3 Division of Biomedical Sciences, St George's, University of London, Cranmer Terrace, London SW17 0RE, UK; 4 Department of Cell Biology & Anatomy and Psychiatry, The Hotchkiss Brain Institute, University of Calgary, Calgary, Canada

**Keywords:** Depression, Systolic blood pressure, Diastolic blood pressure, Cardiovascular risk, Anandamide, 2-arachidonoylglycerol

## Abstract

**Background:**

Depression is known to be a risk factor for cardiovascular diseases but the underlying mechanisms remain unclear. Since recent preclinical evidence suggests that endogenous agonists of cannabinoid receptors (endocannabinoids) are involved in both cardiovascular function and depression, we asked whether endocannabinoids correlated with either in humans.

**Results:**

Resting blood pressure and serum content of endocannabinoids in ambulatory, medication-free, female volunteers with depression (n = 28) and their age- and ethnicity-matched controls (n = 27) were measured. In females with depression, both diastolic and mean arterial blood pressures were positively correlated with serum contents of the endocannabinoids, *N*-arachidonylethanolamine (anandamide) and 2-arachidonoylglycerol. There was no correlation between blood pressure and endocannabinoids in control subjects. Furthermore, depressed women had significantly higher systolic blood pressure than control subjects. A larger body mass index was also found in depressed women, however, it was not significantly correlated with serum endocannabinoid contents.

**Conclusions:**

This preliminary study raises the possibility that endocannabinoids play a role in blood pressure regulation in depressives with higher blood pressure, and suggests an interrelationship among endocannabinoids, depression and cardiovascular risk factors in women.

## Background

Recently, the lipid signaling molecules endocannabinoids (eCBs) have been linked to depression and anxiety disorders [1-5]. The eCBs are endogenous agonists of cannabinoid CB_1 _and/or CB_2 _receptors and the prototypical examples are *N*-arachidonylethanolamine (anandamide; AEA) and 2-arachidonoylglycerol (2-AG). These hydrophobic molecules are produced 'on demand' in the central and peripheral system by stimuli, such as depolarization, increases in intracellular Ca^2+ ^and activation of G protein-coupled receptors linked to phospholipase cascades [6,7]. Interestingly, cannabinoid CB_1 _receptor knock-out mice display biochemical and behavioral changes that are often seen in depression, including increased activity of the hypothalamus-pituitary-adrenal axis, anxiety, anhedonia, reduced feeding and weight loss [2,8,9]. Alterations in eCB contents and/or CB_1 _receptor signaling have also been associated with stress and depression in laboratory animals, depending on the context, stressor and brain region [3-5]. It remains to be established whether or not pathological changes in eCB contents worsen or alleviate symptoms of depression. In an attempt to investigate this, we have previously examined the serum content of eCB in women diagnosed with depression [10,11]. We observed that serum content of AEA and 2-AG was increased in minor depression but decreased in major depression, suggesting that the endocannabinoid system may be disturbed in depressive disorders. There is also indication that circulatory eCBs levels are correlated with exposure to stress [11], anxiety ratings and duration of depressive episode [10]. The role of eCBs in depression is further supported by the recent findings that variations of the gene encoding CB_1 _receptors is associated with depressive symptoms [12] and a reduction in antidepressant treatment response in major depression, especially in women with high anxiety level [13].

Depression is not only a persistent mood disorder, but also a risk factor for cardiovascular diseases. It is prevalent in heart failure patients and predicts poor prognosis for those with ischemic heart disease or following myocardial infarction [14,15]. The underlying mechanisms of this association are unclear but the higher occurrence of hypertension and changes in cardiac and arterial functions in depressed patients could play a role. For instance, depression has been associated with impaired autonomic control, enhanced platelet reactivity, upregulation of proinflammatory mediators and development of atherosclerotic plaques [14-17]. AEA and 2-AG are known to modulate cardiovascular functions [18]. In rodents, AEA and/or 2-AG can cause arterial dilation [19,20], reduced cardiac contractility [21] and hypotension [22,23]. Furthermore, AEA and other CB_1 _receptor agonists modulate autonomic transmission [24,25], platelet reactivity [26,27] and inflammatory processes [28], all of which have been implicated in the cardiovascular risks associated with depression in humans. These data lead us to hypothesize that eCBs are involved in the interaction between depression and cardiovascular function. Using the same cohort of volunteers as in our recent study [10], we have examined the relationship between serum eCB content and resting blood pressure in depressed women.

## Results

### Correlations between serum eCBs and blood pressure in depressed individuals

The average systolic blood pressure was significantly higher in depressed individuals than their matched controls (Table 1). There were no significant overall differences in their averaged diastolic pressure, mean arterial pressure or heart rate (Table 1); however further analysis found that these parameters were significantly increased in minor as compared to major depression (Table 1).

**Table 1:** Cardiovascular parameters and body mass index

	MAP (mmHg)	SBP (mmHg)	DBP (mmHg)	HR (beats/min)	BMI (kg/m^2^)
Control	81.4 ± 1.6	108.6 ± 1.5	67.9 ± 1.5	70.0 ± 1.8	25.4 ± 1.0

Depressed patients	85.6 ± 1.7	115.1 ± 2.1*	69.8 ± 1.3	69.0 ± 2.0	31.5 ± 1.8**

Minor depression	89.7 ± 3.3*†	118.2 ± 4.0*	73.7 ± 2.3*††	71.9 ± 3.2††	31.9 ± 3.2**

Major depression	82.6 ± 1.4	112.9 ± 2.1	66.9 ± 1.2	66.8 ± 2.4	31.2 ± 2.2**

* *P *< 0.05, ** *P *< 0.01 vs control; † *P *< 0.05, †† *P *< 0.01 vs patients with major depression; MAP, mean arterial blood pressure; SBP, systolic blood pressure; DBP, diastolic blood pressure; HR: heart rate; BMI, body mass index

**Figure 1:**
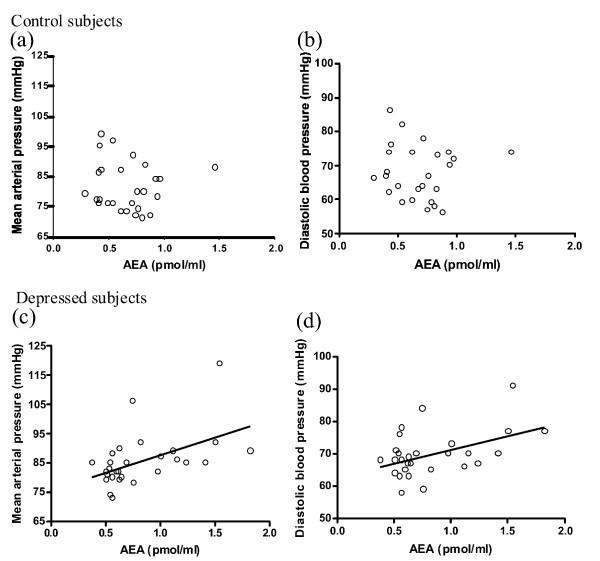
**Scatter plots of serum content of *N*-arachidonylethanolamide (AEA) and mean or diastolic blood pressure in control (a, b) or depressed subjects (c, d)**. n = 27-28. Serum AEA is positively correlated with mean (r = .50) and diastolic blood pressure (r = .46) in depressed subjects (c.f. Table 2).

**Figure 2:**
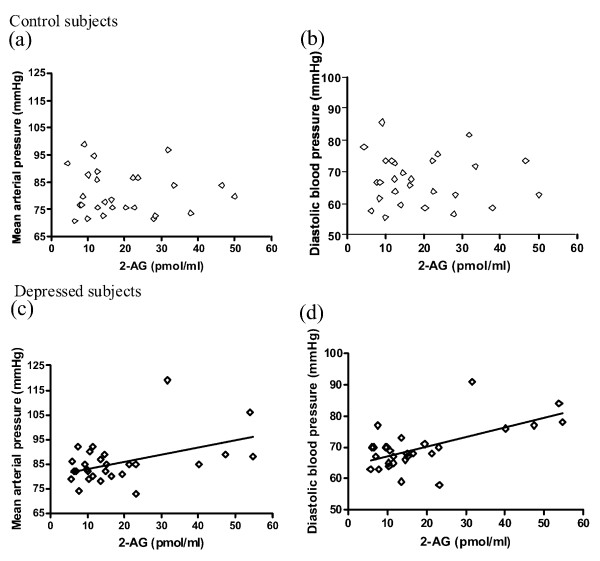
**Scatter plots of serum content of 2-arachidonoylglycerol (2-AG) and mean or diastolic blood pressure in control (a, b) or depressed subjects (c, d)**. n = 27-28. Serum 2-AG is positively correlated with mean (r = .46) and diastolic pressure (r = .62) in depressed subjects (c.f. Table 2).

Pooling data from all depressed subjects masked the bidirectional changes in serum eCB contents in minor versus major depression [10], such that the averaged serum AEA and 2-AG contents were similar in depressed women (AEA: 0.8 ± 0.1 pmol/ml; 2-AG: 18.5 ± 2.7 pmol/ml) and in control subjects (AEA: 0.7 ± 0.1 pmol/ml; 2-AG: 19.0 ± 2.4 pmol/ml). However, we found that both serum AEA and 2-AG were positively correlated with diastolic and mean arterial pressure in the combined group of women diagnosed with major and minor depression (Table 2). Based on Pearson's Correlation tests (r), it was estimated that about 20% to 38% of variance in eCB content could be explained by variation in diastolic pressure or mean arterial pressure in women with depression, and *vice versa*. In addition, AEA tended to correlate positively with systolic pressure or heart rate (Table 2); it might account for > 11% of the variance in systolic pressure and heart rate. In contrast, no correlations between serum eCBs and blood pressure or heart rate were found in the control subjects (Table 2). The distinct distributions of serum eCBs in relation to blood pressure readings in control and depressed subjects are shown in Figure 1 (for AEA) and Figure 2 (for 2-AG). Importantly, significant correlations between eCBs and diastolic pressure, and between eCBs and mean blood pressure, were also obtained after controlling for a third variable; use of tobacco (% daily smokers, Depressives, 25%; Control: 0%), use of oral contraceptives (% user, Depressives, 18%; Control: 30%), alcohol consumption (number of alcoholic drinks per week, Depressives, 2.6 ± 0.9; Control, 1.3 ± 0.7), BMI (see below), waist-to-hip ratio (see below) or serum total cholesterol levels (data not shown).

**Table 2:** Correlation between serum endocannabinoids and blood pressure in depression

		AEA		2-AG	
		r*	**P†**	**r**	**P**

Control n = 27	MAP	-.07	0.72	-.06	0.78
	
	SBP	-.17	0.40	.04	0.83
	
	DBP	-.05	0.81	-.04	0.84
	
	HR	-.02	0.92	-.13	0.54

Depressed patients n = 28	MAP	.50	< 0.01‡	.46	0.01‡
	
	SBP	.35	0.07	.22	0.26
	
	DBP	.46	0.01‡	.62	< 0.01‡
	
	HR	.34	0.08	.14	0.48

* correlation coefficient as determined by Pearson correlation tests; † probability value; ‡ statistical significance; MAP, mean arterial blood pressure; SBP, systolic blood pressure; DBP, diastolic blood pressure; HR: heart rate

BMI values were significantly elevated in depressed women (Table 1). It was noted that 2 of the depressed patients would be considered for hypertensive treatment (with systolic/diastolic: 152/91 and 140/84 mmHg) and had a BMI of 59 and 29 kg/m^2 ^respectively. In both the control and depressed subjects, BMI was positively correlated with systolic pressure (Depressive: r = .41, *P *= 0.03; Control: r = .38, *P *= 0.05) but not diastolic pressure (Depressive: r = .27, *P *= 0.17; Control: r = -.01, *P *= 0.97). In depressed subjects, BMI was also positively correlated with mean arterial pressure (Depressive: r = .40, *P *= 0.03; Control: r = .15, *P *= 0.46). However, BMI was not correlated with serum eCBs in either group (Depressives, AEA: r = .28, *P *= 0.15; 2-AG: r = .03, *P *= 0.88; Control, AEA: r = -.02, *P *= 0.93; 2-AG: r = .05, *P *= 0.82).

As in the case for BMI, waist-to-hip ratio, which is an indicator of abdominal obesity, of depressed subjects (0.82 ± 0.01) was also significantly larger than that of control subjects (0.79 ± 0.01; *P *= 0.01). In control subjects, 2-AG was significantly and positively correlated with waist-to-hip ratio but no other correlations were significant (Depressives, AEA: r = -.03, *P *= 0.90; 2-AG: r = .23, *P *= 0.24; Control, AEA: r = .28, *P *= 0.17; 2-AG: r = .41, P = 0.03).

Total cholesterol levels in serum were not different between controls (183.1 ± 6.9 mg/dL) and depressed subjects (180.7 ± 5.8 mg/dL). There was a tendency for 2-AG, but not AEA, to be positively correlated with serum cholesterol in both groups (Depressives, AEA: r = .08, *P *= 0.68; 2-AG: r = .33, *P *= 0.09; Control, AEA: r = .03, *P *= 0.90; 2-AG: r = .33, *P *= 0.09). No correlation between cholesterol and blood pressure was detected (data not shown).

### Correlations between serum eCBs and blood pressure in major versus minor depression

We further examined if correlations between serum eCBs and blood pressure reported herein depend on the severity of depression. The mean arterial (*P *= 0.04) and diastolic blood pressure (*P *= 0.01) in minor depression was significantly greater than that in major depression (Table 1).

In subjects with minor depression, 2-AG, but not AEA, was strongly and positively correlated with diastolic blood pressure (Table 3). Based on Pearson's Correlation tests, it was estimated that almost 51% of variance in 2-AG content could be explained by variation in diastolic pressure and *vice versa*. In addition, AEA was positively correlated with heart rate in minor depressives, accounting for about 32% of variance (Table 3). In subjects with major depression, AEA, but not 2-AG, was positively correlated with diastolic pressure (Table 3). It was estimated that about 23% to 29% of variance of AEA content could be explained by variation in diastolic pressure and *vice versa*. AEA also tended to correlate positively with mean arterial blood pressure (Table 3). The correlations between eCBs (AEA or 2-AG) and diastolic pressure remained significant after controlling for a third variable, including: use of tobacco, use of oral contraceptives, alcohol consumption, BMI, waist-to-hip ratio or serum total cholesterol levels (data not shown).

**Table 3:** Correlation between serum endocannabinoids and blood pressure in minor versus major depression

		AEA		2-AG	
		r*	**P†**	**r**	**P**

Minor depression n = 12	MAP	.44	0.15	.44	0.15
	
	SBP	.36	0.25	.22	0.49
	
	DBP	.30	0.34	.71	< 0.01‡
	
	HR	.57	0.05‡	.11	0.74

Major depression n = 16	MAP	.48	0.06	-.20	0.47
	
	SBP	.21	0.43	-.23	0.40
	
	DBP	.54	0.03‡	-.30	0.26
	
	HR	-.06	0.84	-.22	0.41

In the same cohort of females, serum AEA is significantly increased in minor depression, whereas 2-AG is decreased in major depression ^11^. * correlation coefficient as determined by Pearson correlation tests; † probability value; ‡ statistical significance; MAP, mean arterial blood pressure; SBP, systolic blood pressure; DBP, diastolic blood pressure; HR: heart rate

BMI values were significantly increased in both minor and major depression (Table 1). Interestingly, BMI was positively correlated with systolic and mean arterial blood pressure in individuals with minor (MAP: r = .63, *P *= 0.02; SBP: r = .59, *P *= 0.03), but not major (MAP: r = .00, *P *= 0.99; SBP: r = .09, *P *= 0.73), depression. BMI was not significantly correlated with diastolic pressure (Minor depression, r = .44, *P *= 0.15; Major depression, r = .02, P = 0.95). In addition, no correlation between eCBs and BMI was detected in either depression group (Minor depression, AEA: r = .39, *P *= 0.21; 2-AG: r = -.04, *P *= 0.89; Major depression, AEA: r = .13, *P *= 0.64; 2-AG: r = .19, *P *= 0.48). It was noted that there was a tendency for 2-AG, but not AEA, to be positively correlated with serum cholesterol in minor depression (Minor depression, AEA: r = -.05, *P *= 0.89; 2-AG: r = .57, *P *= 0.06; Major depression, AEA: r = .10, *P *= 0.70; 2-AG: r = -.05, *P *= 0.86).

## Discussion

The major finding in this study was that serum content of both AEA and 2-AG were positively correlated with both diastolic and mean arterial pressure in ambulatory, medication-free, women with depression. We also found that diastolic pressure was positively correlated with 2-AG in minor depression but with AEA in major depression, hinting at a differential role of the eCBs in depression. Our data also confirm previous suggestions that depression is accompanied by elevated blood pressure, which is a major risk factor for cardiovascular diseases. To our knowledge, this is the first report of a correlation between eCBs and blood pressure in clinical depression. This correlation, however, does not necessarily imply a casual relationship. It is also important to note that it is too early to generalize our data to a larger population. Notably, the current data were obtained in relatively young, female volunteers. Therefore it remains to be determined if similar results also occur in older subjects and in men.

By still unknown mechanisms, depression seems to have a negative impact on basic cardiovascular functions. For instance, reductions in heart rate variability [15] and baroreflex sensitivity [17] as well as increases in blood pressure or hypertension [29] have been reported in depressed patients. These changes might explain why depression is a risk factor for cardiovascular diseases, and increases mortality and morbidity in patients with myocardial infarction or heart failure [14,15]. In this study, we found that depressed women have significantly higher systolic pressure (by an average of 6.5 mmHg) and also tend to have a higher mean arterial pressure (by an average of 4.2 mmHg). Interestingly, increases in systolic and mean arterial blood pressure are more evident in individuals with minor, as compared to major, depression. For the majority of depressed subjects, their systolic and diastolic pressures remain within the normotensive range [30]. Nevertheless, it is noteworthy that several epidemiological studies have suggested a continual and gradual increase in cardiovascular risks with increased systolic and diastolic blood pressure, even when pressures remain within normal range [31,32].

There is strong evidence from laboratory animals that eCBs are involved in the regulation of blood flow and blood pressure, especially in pathophysiological conditions such as hypertension [18]. Exogenous addition of AEA or 2-AG can reduce cardiac contraction, vascular tone and arterial blood pressure [19-23]. Importantly, Kunos and colleagues [22,33,34] have demonstrated that the hypotensive effects of AEA are more pronounced in hypertensive, anaesthetized animals. Indeed, in Spontaneously Hypertensive Rats, a commonly used model for genetic hypertension, inhibition of enzymatic degradation of endogenous AEA is sufficient to reduce blood pressure [33]. Notably, modulation of eCB signaling, by using AEA enzyme inhibitors or CB_1 _receptor antagonist, regulates blood pressure in hypertensive, but not normotensive rats. These results suggest that an eCBs tone limits the extent of hypertension under pathophysiological conditions. This might explain our observed correlations of eCBs and blood pressure in the depressed (hypertensive) but not in healthy subjects. On the other hand, increases in blood pressure have recently been shown to elevate AEA content in the regulatory center for baroreceptor reflex [35]. It has been proposed that AEA enhances baroreflex function, which might be compromised in depression [17], by increasing neuronal activity at the nucleus tractus solitarius [35,36].

Our data that serum contents of AEA and 2-AG are strongly and positively correlated with diastolic and mean arterial blood pressure in depressed but not healthy women, suggest that serum eCBs could function to compensate for an elevated blood pressure in depression. If this is true, the data also suggest that this feedback mechanism fails to normalize the systolic pressure, which remains elevated in depressives. This hypothesis is consistent with the animal data available; however, the possibility that eCBs somehow contribute to the increase in blood pressure cannot be ruled out. It is interesting that serum eCB content and blood pressure are not correlated in control subjects. Furthermore, despite opposing changes of averaged, serum eCB content in minor and major depression [10], either AEA or 2-AG remains positively correlated with diastolic blood pressure in both depression groups. Taken together, the present findings suggest that a positive correlation between serum eCBs and blood pressure could be a common feature of depression at different levels of severity. It is conceivable that biochemical changes in depression, or its associated increase in blood pressure, play a facilitatory role in the positive correlation between eCBs and blood pressure.

Emerging evidence suggests changes in eCB signaling are involved in depression and anxiety disorders. In the same cohort of female subjects as in the current study, we observed that serum 2-AG content is negatively correlated with the duration of current depressive episode and AEA content is negatively correlated with Hamilton ratings for cognitive and somatic anxiety in depressed subjects [10]. However, there is no correlation between these parameters and blood pressure (data not shown). Together, our results point to the complex interrelationships among serum content of eCBs, depression and blood pressure. In this study, there is also evidence that AEA and 2-AG could play a differential role in minor vs major depression since diastolic pressure was positively correlated with 2-AG in minor depression and with AEA in major depression. The synthesis and degradation of the two eCBs involve distinct enzymes [6,7] and eCB levels are often differentially regulated in stress and anxiety [3,10,11]. Alterations of eCBs signaling in depression and hypertension of different severity remain unclear but our data might indicate that 2-AG is the predominant eCB involved in blood pressure control in minor depression, whereas AEA plays a more important role in major depression. Such differential roles also highlight the need for measurements of both eCBs in future studies on subtypes of depression.

Depression is sometimes associated with an increased prevalence of obesity and smoking, which could act as confounding factors in the association between blood pressure and eCBs. In this study, depressed subjects are more likely to be obese (with BMI ≥ 30 kg/m^2^) compared to their matched controls. Whilst this might contribute to the increased blood pressure in depressives, a direct link between BMI and serum eCBs is not evident. In addition, tobacco smoking, the waist-to-hip ratio, serum total cholesterol also fail to explain the correlations between eCBs and blood pressure in depression. It is noteworthy that the absence of correlation between BMI and eCBs in the current study (in women; average age of 29 years) contrasts with the observation that BMI is positively correlated with plasma 2-AG, but not AEA, in men with an average age of 42 years [37]. The discrepancy could also be due to the limited number of subjects with advanced obesity in our sample [38]. Interestingly, however, there is indication that 2-AG, but not AEA, is positively correlated with serum total cholesterol levels in our female subjects. Since subfractions of cholesterol content were not determined in this study, it is unknown if high-density lipoproteins (HDL)-cholesterol or low-density lipoproteins (LDL)-cholesterol, or both, are involved. This is of particular interest in light of the finding that the CB_1 _receptor antagonist, rimonabant, improves serum HDL-cholesterol of overweight or obese subjects in a randomized, double-blinded clinical trial [39]. Further analysis also revealed that correlations between 2-AG and total serum cholesterol, and between BMI and blood pressure, occurred predominantly in individuals with minor depression. At present, the significance of these subtle differences of cardiovascular and metabolic variables between minor and major depression is unclear.

To conclude, our study shows that serum contents of eCBs are positively correlated with blood pressure in depressed women but not in their matched control subjects. We speculate that eCBs play a role in regulation of blood pressure in depression, perhaps acting to buffer an increase in blood pressure. In this study, depressed women also have a higher systolic blood pressure, body mass index, waist-to-hip ratio and higher prevalence of tobacco smoking, all of which are associated with increased risk of cardiovascular diseases. Since eCBs are likely involved in both cardiovascular and neuronal functions, the interrelationships among these agents, cardiovascular parameters and depression warrant further attention.

Given the negative impact of depression on patients who have ischemic heart disease or have recently had myocardial infarction, it has been suggested that effective treatment of depression can not only improve the quality of life but also the clinical outcome of patients with heart diseases. A few, although not all, clinical trials have indeed shown that treating depression with selective serotonin reuptake inhibitors (SSRI) significantly improve the prognosis of patients recovering from myocardial infarction [40-42]. In this regard, it is interesting to note that inhibitors of fatty acid amide hydrolase have been proposed to reduce the symptoms of anxiety and depression [43,44]. Perhaps increasing eCBs signaling by these inhibitors could also help normalize blood pressure homeostasis in depression. However, this approach could also be detrimental to depressive individuals with obesity-related co-morbidities since a recent clinical study has reported that plasma eCBs is inversely related to coronary blood flow in advanced obesity [38]. More clinical studies examining the role of eCBs in depression and cardiovascular functions are required.

## Methods

### Subjects

A total of 55 ambulatory, adult women from Saint Louis, MO, USA participated in the study; the same cohort of volunteers was used in our recent study [10]. The protocol was approved by the Institutional Review Board of Washington University USA and all subjects provided written informed consent. Depressed subjects (n = 28, age 29.0 ± 1.7) met the diagnostic criteria defined by the forth edition of the Diagnostic and Statistical Manual (DSM-IV) [45] for clinical depression. They included subjects with Major Depressive Episode (n = 16) and Minor Depressive Episode (n = 12). Their matched control subjects (based on age and ethnicity; n = 27, age 28.8 ± 1.6) had no lifetime history of psychiatric illness. Each group had 12 Caucasian, 13/14 African American, 1 Hispanic and 1 Asian subjects. All subjects were in good health, defined as having (a) no history of chronic medical illness, (b) no indications of acute infectious disease at study entry, as evidenced by self-report of symptoms and a normal complete blood count, and (c) no prescribed medication regimen, other than oral contraceptives, in the past 6 months including anti-depressants. Candidates were excluded if they were older than 55; had been pregnant in the past year; were menopausal, postmenopausal, had irregular menses; were undernourished as evidenced by serum albumin ≤ 3.3 g/dL; or with reported abuse of illicit substances including cannabis, cocaine and heroin. More detailed information on the recruitment of volunteers, subsequent assessment of clinical depression and demographic characteristics of the sample population has previously been reported [10].

### Blood pressure measurement

Subjects were seated in a comfortable chair with forearm on the armrest. Using appropriate cuff size, 3 blood pressure readings, spaced 2 min apart, were collected by an automated oscillometric device (Dinamap Pro 100; Critikon Corporation). The averaged systolic, diastolic and mean arterial blood pressure were obtained. This was followed by blood draw, via antecubital venipuncture, in the morning hours (0900 h - 1200 h). Blood samples were centrifuged for 15 min at 1000 g and the serum aspirated and frozen at -70 to -80°C until use. All serum samples were frozen by 120 min following venipuncture. Total serum cholesterol was also assessed using enzymatic methods on a Hitachi 747 instrument (Kyowa Medex) in the Washington University Center for Clinical Studies. Upon completion of the study, participants were compensated US$150. These procedures were approved by the Institutional Review Board of Washington University, USA.

### Detection of serum endocannabinoids

Serum eCBs were extracted by using Bond Elut C18 solid-phase extraction columns (1 ml; Varian Inc, Lake Forest, CA). Serum samples (0.5 ml each) were thawed and made up to 15% ethanol, to which the internal standards [^2^H_8_]-AEA (16.9 pmol) and [^2^H_8_]-2-AG (46.5 pmol) (Cayman Chemicals, Ann Arbor, MI) were added. Samples were vortexed and centrifuged at 1000 × g for 4 min. The supernatant was loaded on C18 columns, which have been conditioned with 1 ml redistilled ethanol and 3 ml of double distilled water (ddH_2_O). The remaining pellet was washed with 100 μl of 15% ethanol and centrifuged again for 3 min. The resulting supernatant was also loaded onto the C18 column. Columns were washed with 5 ml ddH_2_O and eluted with 1 ml of ethyl acetate. The ethyl acetate layer in the resulting elute was removed and dried under N_2_. Lipids in the residual ddH_2_O phase were extracted by mixing with an additional 1 ml of ethyl acetate, which was added to the original ethyl acetate solution. Once dried, samples were resuspended in 20 μl of methanol and stored at -80°C. AEA and 2-AG were quantified using isotope-dilution, atmospheric pressure, chemical ionization liquid chromatography/mass spectrometry (LC-APCI-MS) as described previously [46].

### Data and statistical analysis

We have recently detected a bidirectional change of serum contents of AEA and 2-AG in minor versus major depression in the same cohort of female subjects [10]. To reveal potential, underlying relationship between serum eCB content and resting blood pressure, we therefore first examined data obtained from all depressed subjects (with either major or minor depression). Further analysis was then performed to explore the impact of the severity of depression on correlations between serum eCBs and blood pressure.

Data are given as mean ± SEM. Blood pressure, heart rate and BMI in individuals with depression and their matched controls were compared by Student's unpaired t-tests or one-way analysis of variance (Prism 4.02; GraphPad Software Inc). Correlations between eCB content and blood pressure were analyzed by bivariate, Pearson's correlation tests (SPSS 14.0; SPSS Inc.). Influences of potential confounders on these correlations were tested by controlling for a third variable, for example tobacco use, body mass index (BMI) and serum cholesterol level, using partial correlation tests (SPSS 14.0; SPSS Inc). *P *≤ 0.05 was considered statistically significant.

## Abbreviations

2-AG: 2-arachidonoylglycerol; AEA: Anandamide; BMI: Body mass index; CB_1_: Cannabinoid receptor type 1; eCB: Endocannabinoids; DSM-IV: Diagnostic and Statistical Manual; HDL: High-density lipoprotein; LDL: Low-density lipoproteins.

## Competing interests

The authors declare that they have no competing interests.

## Authors' contributions

MNH, GEM, BBB and CJH designed the study; WSVH, MNH and GEM performed the experiments; WSVH, MNH and CJH wrote the paper. All authors read and approved the final manuscript.
